# Identification and determination of synthetic colors in fine bakery wares (cakes) sold in Riyadh City, Saudi Arabia: a comparative study of the specifications set by the Gulf standardization organization and the European Union

**DOI:** 10.3389/fnut.2026.1787606

**Published:** 2026-03-17

**Authors:** Mazen M. Al Fayez, Khaled I. Alyousef, Bader I. Al Tamran, Fahad N. Abu-Khalil, Faisal H. Alruways, Somaiah K. Almubayedh

**Affiliations:** Saudi Food and Drug Authority (SFDA), Riyadh, Saudi Arabia

**Keywords:** colors, confectionery, fine bakery wares, sweets, synthetic colors

## Abstract

**Introduction:**

Synthetic food colorants are widely used in bakery products to enhance visual appeal; however, their use is regulated due to potential health concerns. This study aimed to identify and quantify synthetic colors in fine bakery wares (cakes) sold in Riyadh City, Saudi Arabia, and to evaluate their compliance with the specifications established by the Gulf Standardization Organization (GSO) and the European Union directive.

**Methods:**

A total of 103 samples were collected from 25 fine bakery brands in Riyadh City, Saudi Arabia. The sampled products included red velvet cake, cupcake, cube cake, mini cake, cheesecake, roll cake, and push-up cake. The analysis of synthetic colorants was performed using reversed-phase High-Performance Liquid Chromatography with a Diode Array Detector (HPLC-DAD). The results were compared with the regulatory limits specified in the Gulf standard (GSO 2500/2015) and the European directive (94/36/EC).

**Results:**

Out of the 103 analyzed samples, 80 samples (78%) contained synthetic food colorants, while 23 samples (22%) showed no detectable colors. Among the positive samples, 18 samples (18%) were non-compliant with the Gulf standard (GSO 2500/2015), whereas 37 samples (36%) were non-compliant with the European directive (94/36/EC). The synthetic colorants investigated included Tartrazine (E102), Quinoline Yellow (E104), Sunset Yellow (E110), Carmoisine (E122), Ponceau 4R (E124), Erythrosine (E127), Allura Red AC (E129), Patent Blue V (E131), Indigo Carmine (E132), Brilliant Blue (E133), Green S (E142), Fast Green FCF (E143), Brilliant Black (E151), and Brown HT (E155). The detection frequencies were 19%, not detected, 3%, 10%, not detected, 3%, 36%, not detected, 2%, 22%, not detected, not detected, 1%, and 3%, respectively. Additionally, an unidentified oil-soluble color was detected in 1% of the samples.

**Discussion:**

The findings indicate the presence of synthetic colorants in a large proportion of bakery products, with a notable number of samples failing to comply with regulatory standards, particularly when evaluated against European limits. These results highlight the need for strengthened regulatory oversight and stricter enforcement of food color regulations to ensure compliance with Gulf standards, safeguard consumer health, and improve the quality and safety of bakery products available in the local market.

## Introduction

In recent years, the number of fine bakery wares shops has increased significantly in Saudi Arabia. The products are produced and sold fresh to consumers with a limited shelf life. Furthermore, most products lack proper labelling in accordance with the Gulf Regulation Number (GSO 9/2013). Food colorants are frequently used in such products to enhance the product’s attractiveness and experience. Food colors are defined as “substances which add or restore color in a food, and include natural constituents of foodstuffs and natural sources which are normally not consumed as foodstuffs as such and not normally used as characteristic ingredients of food” (1). The GSO 9/2013 classifies food colors as food additives. The (GSO standard) defines food colorants as any substance that is not consumed on its own and is not used as an essential ingredient in food, whether or not it has nutritional value. Moreover, food additives are intentionally added to food for technological purposes, such as enhancing color, taste, and/or smell (2). The US Food and Drug Administration (USFDA) defines color additives as “any substance that imparts color to a food, drug, cosmetic, or to the human body” (3).

Food colors are used in food production for several reasons: (1) to balance the color loss as a result of the exposure to moisture, light, abused temperature, and bad storage conditions; (2) to promote and enhance original disparities in the color of the food item; (3) to intense the natural color of a food item; (4) to provide colorful looking to some products such as candies, bakery products, and other types of food (4).

Food colors are classified into two main categories based on their methods of production. The first category comprises naturally extracted substances, whereas the second category comprises synthetically manufactured food colorants (2). Natural colors are extracted from natural living sources such as plants or animals. On the contrary, synthetic colors were once produced from coal tar (5). Currently, synthetic colors are petroleum-based products (6). There are so many natural colors in nature that have been used for a long time, such as Carotenoids, Chlorophyll, Anthocyanins, Turmeric, Carmine, and many others.

The changes that have taken place in food production over the past decades have transformed how food is produced and approached. Thus, food manufacturers have been extensively using synthetic colors for several reasons. First, the low cost of synthetic colors compared to the natural ones is a major drive for food producers. Food producers tend to use materials that provide the required product’s properties and quality at a lower cost. Secondly, the shelf-life of synthetic colors is relatively longer than that of natural ones, which favors the synthetic colors over the natural ones. Lastly, the ability and flexibility to produce different varieties of synthetic colors is easier than producing natural colors. Manufacturers can produce different types of synthetic colors with varying properties, whereas natural colors are limited to the inherent properties of the natural source (7). Synthetic colors can be classified into three categories based on their solubility properties. First, water-soluble colorants, such as *Allura Red AC*, *Amaranth,* and *Sunset Yellow*. Second, oil-soluble colorants. The use of oil-soluble colorants is prohibited in food production due to the toxic effects, which may include hepatotoxicity, carcinogenicity, bioaccumulation in fatty tissues, neurotoxicity, especially in children, and endocrine disruption (8, 24). Third, there are lake colorants that are not soluble in water, oil, or any other solvent. This category disperses in food and thus produces the required color. Lake colorants are used in the production of cakes, biscuit fillings, soups, spice mixes, powder drinks, confectionery, sweets, and powder drinks (5).

Food colors are classified in another way based on their legal use. They are classified as permitted and non-permitted food colorants (9). When non-permitted food colors are used in food production, it is considered an illegal practice that requires certain measures to be taken by the authorities in the country.

There are several colors listed in the database of the Codex Alimentarius Commission; these colors have been evaluated by the Joint FAO/WHO Expert Committee on Food Additives (JECFA) (10). The committee has evaluated and set specifications for each food colorant (11).

In the United States, the USFDA has also approved and listed several colors permitted for use in food production, some of which are synthetic and require a batch certification process before use (12). Color additives are regulated in the US and controlled by the USFDA. The food color undergoes a certification process before being placed in the market for public or commercial use. Colors are classified into two main categories: (1) exempt from certification; (2) subject to certification. Both categories have high standards of criteria and requirements before being approved for marketing. Exempt-from-certification colors are those extracted from natural sources, such as plants and animals. Conversely, certified colors are synthetically produced colors that should undergo a rigorous certification process (13).

In Europe, Directive 94/36/EC has allowed the use of a total of 45 colors in food production, of which 16 are synthetic (4 of which have limited use in food production (14)). The use of food colorants in food production is restricted and controlled in Europe by the European Commission. According to Article 6 in the directives, the member states are obliged to have a system in place to monitor the consumption and the use of colors in food production. Member states must report the findings of the monitoring program to the EU Commission, which then shall report any changes and recommendations to the European Parliament (15).

In Saudi Arabia, the use of food colorants is regulated and monitored by the Saudi Food and Drug Authority (SFDA) in accordance with the Gulf Standard (GSO 2500/2015). SFDA monitors food products through its annual national monitoring program. Results from the program are gathered and analyzed, and then actions are taken when a breach of the standard exists.

Food colorants have gained a controversial debate amongst scientists and regulatory bodies over their health impacts. Contradictory reports and studies have been out to either prove or reject the health impact of food colorants. As illustrated earlier, food colorants can be either natural or synthetic (6). Carotenoids, for example, are natural pigments that have a positive impact on the treatment of arthritis and cancer. Moreover, Anthocyanins have been useful in the treatment of coronary heart disease, hypertension, and liver disorders. Furthermore, another natural color, so-called Betalains, is an antimicrobial, antiviral, and anticarcinogenic. Furthermore, Monascus pigments can be used as anti-tumorigenic and antimutagenic agents. Conversely, synthetic colors have been under frequent assessment and evaluation by many scientists and regulatory bodies in recent years.

In 2008 the Center for Science in the Public Interest (CSPI) in Washington, DC, issued a Petition to the USFDA so called Food Dyes—A Rainbow of Risks to evaluate (9) dyes approved by the USFDA; namely, Blue 1, Blue 2, Citrus red 1, Green 3, Orange B, Erythrosine, Red 40, Yellow 5, and Yellow 6. The report alleged that these colors pose health concerns for the public. Several health-impacting factors have been reviewed, such as carcinogenicity and Neurotoxicity (hypersensitivity reactions and behavioral problems). The report concluded that further safety studies are needed before considering the safety of the dyes (16). The USFDA has appreciated the report prepared by CSPI and stated that these colors are safe based on the available safety data (17).

On the other side of the continent, in 2008, the Panel on Food Additives and Nutrient Sources added to food (ANS)—a scientific committee of EFSA—reviewed the study conducted by researchers from Southampton University on behalf of the UK Food Standards Agency (FSA). The study indicated that “certain mixtures of five azo dyes (the group in Question 3 above, excluding Amaranth), the color Quinoline Yellow (E104) and the preservative sodium benzoate (E211) may have a small effect on activity and attention in certain groups of children”. The (ANS) panel concluded that the findings could not be used to alter the Acceptable Daily Intake (ADIs) for the individual colors being studied. The reason for not accepting the findings of the study was that it assessed the effects of the mixture of the additives and did not look at the effects of the single additive. In 2009, after assessing all of the available data, the (ANS) panel lowered the ADI for three food colorants: Sunset Yellow FCF (E110), Quinoline Yellow (E104), and Ponceau 4R (E124). They concluded that, as a result of exposure, these colors could probably exceed the new ADIs for both kids and adults. Following that, EFSA in 2014 set ADIs of 4 mg/kg body weight for Sunset Yellow FCF. Tartrazine (E102), Azorubine/Carmoisine (E122), and Allura Red AC (E129) ADIs were not changed by the panel and remained the same. The panel concluded that children who consume an uncontrolled amount of commodities containing Azorubine/Carmoisine or Allura Red AC would probably be at risk of exceeding the ADIs (14).

The 2019 update to the technical regulation for synthetic food colors by the SFDA represents a critical step toward ensuring food safety and protecting public health. The revised regulation is based on internationally recognized references such as JECFA and the Codex Alimentarius, ensuring that synthetic colors are used within scientifically established safe limits. It also enhances alignment with Gulf and international standards, facilitating trade and ensuring compliance with local products. Moreover, the update helps manufacturers adhere to Good Manufacturing Practices (GMP) and improves transparency by clearly identifying permitted additives and usage levels. These efforts reflect the SFDA’s ongoing commitment to strengthening regulatory systems and promoting high standards of food quality and safety. This study evaluates compliance with regulatory limits for chemical food additives, with particular emphasis on permitted levels and conformity with established standards.

## Materials and methods

### Reference materials

Reference standards were used with different purities and different manufacturers as following: Tartrazine (E102) (CAS No. 1934-21-0) with 85% purity, Carmoisine (E122) (CAS No. 3567-69-9) with 73% purity, Amaranth (E123) (CAS No. 915–67-3) with 85% purity, Ponceau 4R (E124) (CAS No. 2611-82-7) with 75% purity, Allura Red AC (E129) (CAS No. 25956-17-6) with 80% purity, Patent Blue V (E131) (CAS No. 3536-49-0) with 80.2% purity and Green S (E142) (CAS No. 3087-16-9) with 76% purity. These reference standards were purchased from IPS, Poland. Quinoline Yellow (E104) (CAS No. 8004-92-0) with 75% purity, Erythrosine B (E127) (CAS No. 16423-68-0) with 88% purity, Indigo carmine (E132) (CAS No. 860–22-0) with 86% purity, and Brilliant Black BN (E151) (CAS No. 2519-30-4) with 60% purity were all obtained from Sigma-Aldrich, India. Ponceau 6R (E126) (CAS No. 2766-77-0) with 85% purity and Acid Red 1 (E128) (CAS No. 3734-67-6) with 60% purity were purchased from Sigma- Aldrich, USA. Fast Green FCF (E143) (CAS No. 2353-45-9) with a purity of 96% was purchased from Sigma-Aldrich, UK.

### Chemicals

HPLC grade acetonitrile, methanol (>99.9%) assay, and Glacial Acitic acid (>99.8%) were purchased from Merck, Germany. Ammonium Acetate 98% from Panreac AppliChem, Germany. Ultra-pure water was produced in the lab using a Milli-Q Direct 8 deionization unit from Millipore, France. Ammonium hydroxide solution 25% from BDH, USA.

### Standard preparation

Taking into account the purity of the reference material used, stock standards (2 mg/mL) were prepared individually in water by dissolving 100 mg of the reference material in a 50 mL class A filled with deionized water. An intermediate standard mixture (0.1 mg/mL) was prepared by taking 5 mL of each stock standard into a 100 mL volumetric flask and making up the volume with deionized water. After that, calibration standards were prepared by pipetting 0.1, 0.2, 0.5, and 1 mL from the intermediate mixture into 100 mL volumetric flasks and made up the volume to give concentrations of 0.1, 0.2, 0.5, and 1 mg/L, respectively. Furthermore, concentrations of 5, 10, 20, and 40 mg/L were prepared in 50 mL volumetric flasks by pipetting an amount of 2.5, 5, 10, and 20 mL, respectively.

### Reagents preparation

The mobile phase A (MPA) (100 mM ammonium acetate and 0.1 acetic acid) was prepared by weighing 7.708 ± 0.002 g of ammonium acetate and dissolving it in 700 mL of deionized water, then 1 mL of acetic acid was added to the solution, and the volume was completed to 1 liter (durable for 7 days). Mobile phase B is 50:50 V/V of methanol:acetonitrile. The mobile phase was filtered using a 0.45 μm HVLP filter (Whatman, England) and degassed for 30 min in an ultrasonic bath (Elma, Germany). The extraction solution (ES) was prepared by mixing 100 mL of MPA with 400 mL of deionized water and 1 mL of 25% ammonium hydroxide.

### Sample collection and preparation for analysis

A hundred and three samples were purchased from 25 different fine bakery brands in Riyadh city, Saudi Arabia. The samples were red velvet cake, cupcake, cube cake, mini cake, cheese cake, roll cake, and push-up cake. All collected samples were purchased 2–3 days before analysis and stored at 2–8 °C to avoid deterioration. Brands were coded alphabetically from A to Y for confidentiality.

The 3 g sample was weighed in a 50 mL polypropylene centrifuge tube. Then 25 mL of ES was added. The mixture was vortexed using a vortex mixer (Velp, Canada) for 1 min, then sonicated for 30 min and centrifuged (Hettich, Rotina 46 R, Germany) for 20 min at 8,000 rpm. After that, the supernatant was filtered using a PVDF 0.45 μm syringe filter (HPF Millex, Ireland). The filtrate was transferred into a 2 mL HPLC vial from which 5 μL was injected into the HPLC system.

### Instrumentation

The analysis was performed using High-performance liquid chromatography with a UV detector (HPLC-DAD) from Agilent Technologies, USA. Analytical Separation was performed using Zorbax Eclips XDB-C18 (4.6 × 150 mm, 5 μ, Agilent, USA) and guard column Eclips XDB-C18 (4.6 × 12.5 mm, 5 μn, Agilent, USA) at 40 °C. The chromatographic separation was performed on a 30-min run at a 1.5 mL/min flow rate. Gradient mobile phase pumping was used (97% of MPA at 0 min, 75% of MPA at 8 min, 30% of MPA at 18 min, 30% of MPA at 23 min, and 97% of MPA at 27 min). The detector signals used were 430 nm, 480 nm, 522 nm, 625 nm, and 570 nm.

## Results

The results of the analysis were compared against the Gulf standard (GSO 2500/2015) and the European standard (94/36/EC). Samples were compared against these two standards to assess compliance and determine which standard provides greater public protection. The European standard has a broader range of synthetic food colorants than the Gulf standard. The Gulf standard contains the following synthetic colors (E102, E110, E122, E129, E132, E133, E143, E155), while the European standard contains the following synthetic colorants (E102, E104, E110, E122, E124, E129, E131, E132, E133, E142, E151, E155). The limits of the synthetic colors differ between the two standards. Table 1 illustrates the difference and shows that the European standard has a limit of 200 mg/kg for some individual colorants. Moreover, the total colorant content in a sample shall not exceed 200 mg/kg. Furthermore, some colorants have a specific limit of 50 mg/kg per sample. Conversely, the GSO standard sets different limits for each food colorant; furthermore, the GSO does not specify any specification for the sum of colorants in a sample. That means the compliance of food colorants should be considered for each color as an individual (Table 1).

**Table 1:** Synthetic colors comparison between the GSO and the European standard.

Synthetic color	Limits		
	GSO 2500/2015	94/36/EC (General limits)	94/36/EC (specific limits)
E102	GMP	Individually or in combination, shall not exceed 200 mg/kg	NA
E104	No limit		NA
E110	50 mg/kg		May not exceed 50 mg/kg
E122	GMP		May not exceed 50 mg/kg
E124	No limit		May not exceed 50 mg/kg
E129	300 mg/kg		NA
E131	No limit		NA
E132	200 mg/kg		NA
E133	200 mg/kg		NA
E142	No limit		NA
E151	No limit		NA
E155	GMP		May not exceed 50 mg/kg
E143	100 mg/kg	No limit	No limit

In total, 80 samples (78%) tested positive for food colorants. On the other hand, 23 samples (22%) tested negative for food colorants. Of the total number of samples, 19 samples (18%) showed non-compliance with the Gulf standard (GSO 2500/2015). On the other hand, when applying the European standard (94/36/EC), 37 samples (36%) showed non-compliance (Table 2).

**Table 2:** Compliance according to the Gulf standard and the European standard.

Sample types	No. of samples	Percentage
Total no. of samples	103	
Negatives samples	23	22%
Positives samples	80	78%
Non-compliant samples according to GSO 2500/2015	19	18%
Non-compliant samples according to 94/36/EC	37	36%

**Figure 1:**
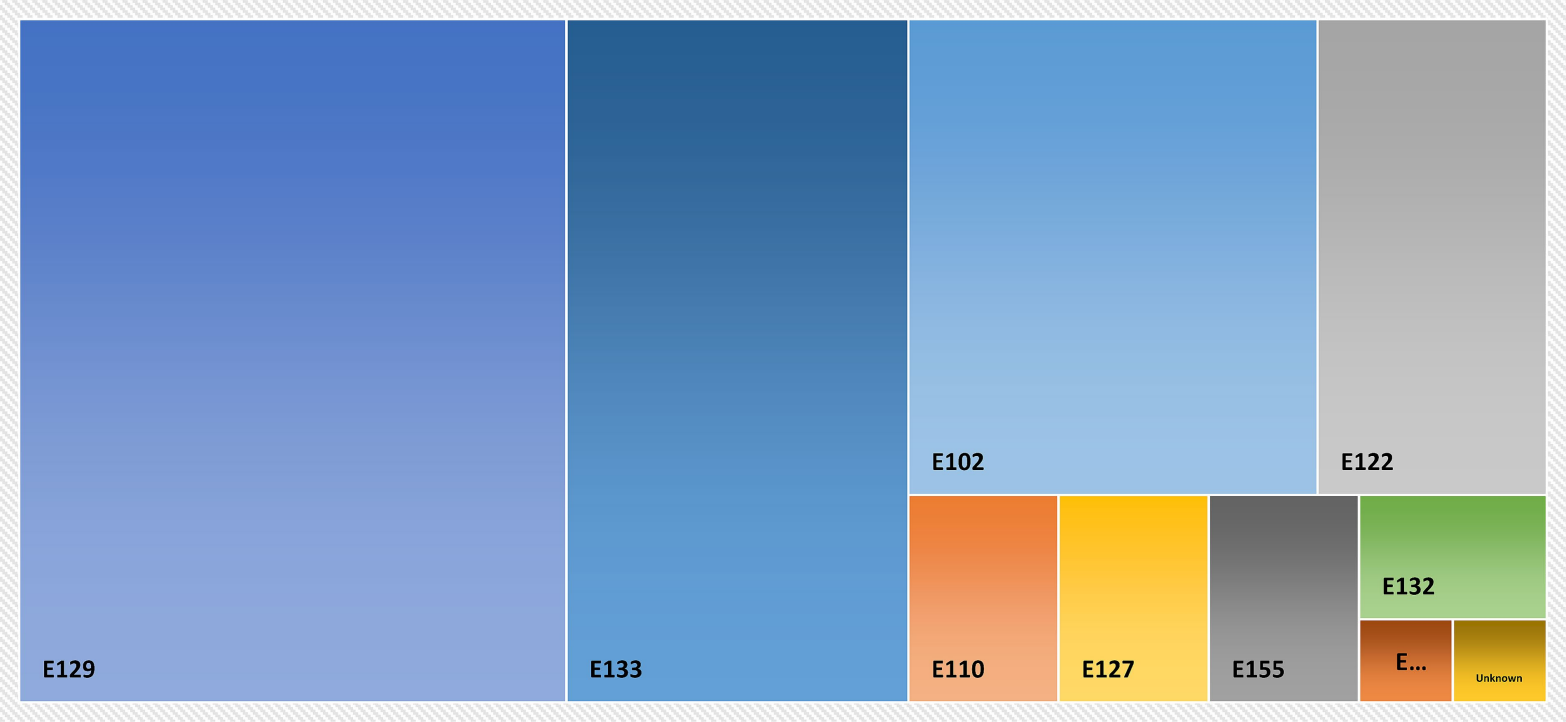
Visual presentation of the frequency magnitude of synthetic food colorants.

Quinoline Yellow (E104), Ponceau 4R (E124), Patent Blue V (E131), Green S (E142), and Fast Green FCF (E143) were not detected in all analyzed samples. Table 3 shows the frequencies and their corresponding percentages of synthetic food colorants detected during the analysis of the samples. Furthermore, it shows the number of non-compliant colors and their corresponding percentages according to the (GSO 2500/2015) and (94/36/EC). Figure 1 shows the synthetic food colorants that were detected most frequently in the analyzed samples.

**Table 3:** The frequency of detection for each color and the non-compliant food colorants according to the GSO and EU standards.

E-number	Name	Color	Samples	Colors frequency		GSO non-compliance		EU non-compliance	
			*n*	*n*	%	*n*	%	*n*	%
E102	(Tartrazine)	Lemon yellow	103	25	19%	GMP^*^	NA^**^	5	5%
E104	Quinoline Yellow	Yellow		0	0	NA^**^	NA^**^	NA^**^	NA**
E110	(Sunset Yellow)	Yellow–orange		4	3%	2	2%	2	2%
E122	Carmoisine (azorubine)	Red to maroon		14	10%	GMP^*^	NA^**^	7	7%
E124	Ponceau 4R	Red		0	0	NA^**^	NA^**^	NA^**^	NA**
E127	Erythrosine	Red		4	3%	2	2%	2	2%
E129	Allura Red AC	Red		48	36%	12	12%	18	17%
E131	Patent Blue	Blue		0	0	NA^**^	NA^**^	NA^**^	NA**
E132	Indigo carmine (indigotine)	Indigo		3	2%	0	0%	0	0%
E133	Brilliant Blue	Reddish blue		30	22%	1	1%	1	1%
E142	Green S	Green		0	0	NA^**^	NA^**^	NA^**^	NA**
E143	Fast Green FCF	Green		0	0	NA^**^	NA^**^	NA^**^	NA**
E151	Black PN, Brilliant Black BN	Black		1	1%	NA^**^	NA^**^	NA^**^	NA**
E155	Brown HT (chocolate brown HT)	Brown		4	3%	NA^**^	NA^**^	NA^**^	NA**
Unknown		Blue		1	1%	NA^**^	NA^**^	NA^**^	NA**
				134	100%				

^*^GMP, Good Manufacturing Practices; ^**^NA, Not Applicable.

Tartrazine (E102) was detected 25 times (19%) in the analyzed samples. The color has no specification in the Gulf standard, as it requires only good manufacturing practices; thus, no conclusion can be drawn. On the other hand, five samples (5%) were non-compliant when applying the European standard. Sunset Yellow (E110) was detected four times (3%) in the analyzed samples. Two samples (2%) were found non-compliant according to the Gulf and European standards, indicating agreement between the two standards. Moreover, Carmoisine (E122) was identified 14 times (10%) in the samples. The Gulf standard has no limits for this color; however, it requires adherence to good manufacturing practices. On the other hand, when applying the European standard, the color was found non-compliant in 7 samples (7%).

Erythrosine (E127) was detected four times (3%) in the samples. According to Gulf and European standards, this color is permitted for use in cherry products. Erythrosine (E127) was non-compliant in 2 samples (2%) for both the Gulf and the European standard. Furthermore, Allura red AC (E129) was identified 48 times (36%) in the samples as the most frequent food colorant. The color was found non-compliant 12 times (12%) according to the Gulf standard. On the contrary, Allura red AC (E129) was found to be non-compliant 18 times (17%) according to the European standard. Indigo carmine (E132) was detected 3 times (2%) in the analyzed samples. All analyzed samples fully complied with this color according to both the Gulf and European standards. Furthermore, brilliant blue (E133) was detected 30 times (22%) in the analyzed samples. Only one sample (1%) was non-compliant to specifications set by the Gulf and the European standard. Moreover, Brilliant black (E151) was found once (1%) in the analyzed samples. The current method used for analysis determines this color as a qualitative result; therefore, no compliance with both standards can be concluded. Brown HT (E155) was detected four times (3%) in the analyzed samples. Furthermore, due to the limitation of the method in quantifying the compound, no conclusion can be drawn regarding the Gulf and European standards. Finally, one sample was found to contain an unknown color, which appeared once (1%).

## Discussion

The results show that 80 samples (78%) were positive for synthetic food colorants and 23 samples (22%) were negative. In a similar study conducted in Jaffna District in Sri Lanka, they found that (93%) of confectionery samples had synthetic food colorants (18). Another study was conducted in Kashan City, Iran, to assess the use of synthetic colors in meat products, sweets, drinks, and miscellaneous foods. The results showed that 72 samples (48.30%) had no colors and 77 samples (51.7%) contained artificial colors. Sweet samples were the most products containing food colorants (72.7%), drinks (51.2%), and meat products (48.10%) (19). Another similar study was conducted to determine the consumption of products containing food colorants during a festival in Hyderabad, India. They found that (44%) consumed sweetmeat products that contained tartrazine and sunset yellow as the most (20). Another study was conducted in Pakistan to evaluate the use of food colorants in 73 samples of sweetmeats and confectionery. They concluded that (58.8%) contained permitted food colorants within the regulatory limit, while (41.1%) of samples were above the regulatory limit. On the other hand, (46.57%) of the samples had non-permitted food colorants (21). Another Assessment study was conducted on Food Stuffs Produced in confectioneries and restaurants in Arak, Iran, to identify the presence of synthetic colors in 70 confectionery samples. The researchers found that 56 samples (80%) violated the regulatory standard (22). Another similar study assessed the use of synthetic colors in pastry, poolak (a type of coin-shaped candy), and rock candy. The results demonstrated that (48.47%) of the samples were positive for food colors and (6.52%) contained illegal colors (23).

The European directive (94/36/EC) imposes stricter compliance requirements than the Gulf standard (GSO 2500/2015) regarding Tartrazine and *Carmoisine* (azorubine). Results showed that the Tartrazine color was not compliant according to the European directive in (5%) of the samples, while the GSO standard required a good manufacturing practice only; thus, all samples complied according to the GSO standard. Similarly, Carmoisine (azorubine) was found non-compliant in (7%) of the samples according to the European directive, while again the GSO standard required good manufacturing practice. The weak criteria set by the GSO may lead to irresponsible use by the manufacturer and producers. It may not help official control inspectors to evaluate the results of these two colors when samples are taken for analysis. Rezaei et al. (22) found that Tartrazine and Carmoisine (azorubine) violation rate according to the Iranian standard was (57.1%) and (28.57%) respectively. Farzianpour et al. (23) found that Tartrazine and Carmoisine (azorubine) were out of legislative limits by (5.3%) and (1.3%) respectively.

Sunset Yellow, Indigo carmine (indigotine), and Brilliant Blue had similar non-compliance rates under the two standards. This is because the specified limits for all colors are the same in both standards. Sunset Yellow is permitted for use in the Gulf and European standards for foodstuffs, with a limit of 50 mg/kg. On the other hand, Indigo carmine (indigotine) and Brilliant Blue are permitted for use in foodstuffs under the Gulf and European standards, with a limit of 200 mg/kg. Sunset Yellow and Indigo carmine (indigotine) appeared to be used less frequently by producers (3 and 2%), with a higher rejection rate for Sunset Yellow (2%), 50% of the samples, and a satisfactory compliance rate for Indigo carmine (indigotine). Although Brilliant Blue is the second color in terms of frequent use by producers, it has shown a high compliance rate (99%). This color seems to be used properly by fine bakery product manufacturers.

According to Gulf and European standards, Erythrosine color is allowed for use in cherry products only. It was found that Erythrosine was used in the production of bakery products, which violates the legislation. The presence and the use of Erythrosine in production support the idea that such practices are not well controlled and managed by producers. The production of such types of foods in bakery retail depends on the ultimate required properties. Thus, limited instruction and documentation for use exist, and poor monitoring is evident. Moreover, and most importantly, the staff making fine bakery products are less educated and trained. They typically gain the experience of producing fine bakery products through practice. This is another reason why tougher monitoring and control, either by producers or by officials, shall be taken for such products.

Allura red AC was detected in 48 samples (36%) as the most frequent color among the others. Allura red AC has a stricter specification limit in the European directive (200 mg/kg) than the Gulf standard (300 mg/kg). As a result, the Allura red was non-compliant against the European directive in (17%) of the samples compared to the Gulf standard (12%). The difference in rejection and acceptance rates between the two standards. Researchers (19) conducted a study to determine the synthetic colors in some locally available foods in Kashan City, Iran. They found that Allura red was not detected in any of the sweet samples (33). A similar study (Dilrukshi et al. 18) conducted to identify synthetic colors in selected confectioneries and beverages in Jaddna District, Sri Lanka. Allura red was not detected in any of the samples (110). Another study (22) conducted to assess the presence of synthetic dyes in foodstuffs produced in confectioneries and restaurants in Arak, Iran. Allura red was detected by (2.85%) of the samples (70)

Black PN, Brilliant Black BN, and Brown HT were assessed in this study as a qualitative measure due to the method’s capability. Black PN and Brilliant Black BN were detected only once, whereas Brown HT was detected four times in the samples. Black PN and Brilliant Black BN have no specification limit in the Gulf standard, whereas the limit set by the European directive is 200 mg/kg. This means this color will not be monitored in Saudi Arabia, and no actions will be taken by the local authorities.

On the other hand, the Gulf standard requires good manufacturing practices (GMP) for Brown HT, while the European directive has set a limit of 200 mg/kg.

**Figure 2:**
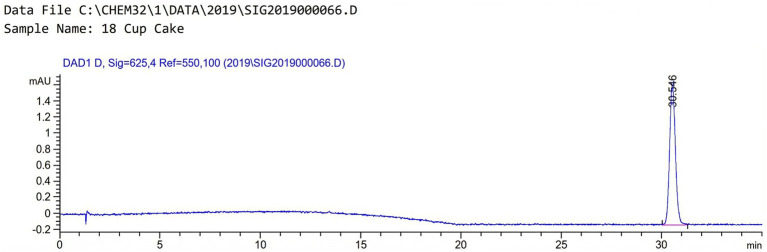
HPLC chromatogram of sample “18 Cup Cake”; detector response (mAU) vs. retention time (min) showing a single peak at 30.546 min.

Finally, unknown colorants were detected in a purple cupcake sample. Further investigations were carried out to identify the color of the substance, which was characterized as blue. After extraction, the oily portion of the sample’s extract appeared blue. That led to the suspicion that an oil-soluble color could be present. Therefore, the oily portion of the sample was collected and diluted with hexane and acetonitrile to identify the color. The diluted sample was injected, and the run time was prolonged to 45 min. The suspected color was detected at 30.5 min (see Figure 2), indicating that an oil-soluble colorant is present. Since oil-soluble colors are prohibited as additives in food processing (22), further investigation may be required to ensure compliance with the legislation.

## Conclusion

In this study, substantial differences were identified between the GSO/2500 standard and European legislation (94/36/EC) regarding the regulation of food colorants. Five food colorants are not regulated under the GSO standard compared with European legislation, whereas one colorant is regulated by GSO but not permitted under EU legislation. Moreover, three food colorants are regulated under the GSO/2500 standard, which is based on Good Manufacturing Practice (GMP), potentially leading to insufficient control and excessive use during manufacturing. Overall, the European legislation appears to apply more stringent regulatory limits. Therefore, revising the current GSO limits, either on an individual colorant basis or considering combined exposure, is recommended. Furthermore, the inclusion and periodic updating of additional colorants not currently covered by the GSO standard should be considered to enhance consumer safety.

Based on these findings, further studies on the use of food colorants and on assessing their potential risks to consumers are essential. As the samples analyzed in this study were collected from private bakeries within a local region, the results highlight the importance of strengthening inspection and monitoring efforts in confectionery and bakery establishments, as this category showed the highest prevalence of synthetic and non-permitted colorants. In parallel, it is recommended that the confectionery and bakery sectors cooperate in providing formal, documented training programs for employees on the correct and safe use of food colorants and their maximum permitted limits, particularly since practical experience is often gained without sufficient scientific or regulatory background. Furthermore, developing consumer awareness programs and public campaigns is advised to inform the public about the risks associated with the use of non-permitted colorants or excessive consumption.

The present study focused specifically on the chemical detection and characterization of food colorants. Microbiological parameters and physical or physicochemical quality attributes such as pH, water activity, moisture content, texture, color stability, and sensory properties were beyond the scope of this investigation. They may be addressed in future research to provide a more comprehensive evaluation of food safety and product quality. Overall, this study contributes to the chemical safety assessment of food colorants and supports regulatory review and risk-based control strategies in bakery products.

## Data Availability

The data are not publicly available due to commercial confidentiality and regulatory considerations but are available from the corresponding author upon reasonable request. Requests to access the datasets should be directed to skmubayedh@sfda.gov.sa.
